# Examining critical factors affecting graduate retention from an emergency training program in Addis Ababa, Ethiopia: a qualitative study of stakeholder perspectives

**Published:** 2017-04-20

**Authors:** Meredith Kuipers, Amira Eapen, Joel Lockwood, Sara Berman, Samuel Vaillancourt, James Maskalyk, Aklilu Azazh, Megan Landes

**Affiliations:** 1 University Health Network, Toronto, Ontario, Canada; 2 Global Health Program, McMaster University, Ontario, Canada; 3 Department of Medicine, Division of Emergency Medicine, University of Toronto, Ontario, Canada; 4 St. Michael’s Hospital, Toronto, Ontario, Canada; 5 Deparment of Emergency Medicine, College of Health Sciences, Addis Ababa University, Addis Ababa, Ethiopia; 6 Department of Family & Community Medicine, Division of Emergency Medicine, University of Toronto, Ontario, Canada

## Abstract

**Background:**

In Ethiopia, improvement and innovation of the emergency care system is hindered by lack of specialist doctors trained in emergency medicine, underdeveloped emergency care infrastructure, and resource limitations. Our aim was to examine the critical factors affecting retention of graduates from the Addis Ababa University (AAU) post-graduate emergency medicine (EM) training program within the Ethiopian health care system.

**Methods:**

One post-graduate trainee and one program manager from the AAU and the University of Toronto (UT) partnership conducted qualitative interviews with current AAU EM residents and stakeholders in Ethiopian EM. Qualitative inductive thematic analysis was performed.

**Results:**

Resident and stakeholder participants identified critical factors in three domains: the *individual condition,* the *occupational environment,* and the *national context.* Within each domain, priority themes emerged from the responses, including the importance of career satisfaction over the career continuum (individual condition), the opportunity to be involved in the developing EM program and challenges associated with resource, economic, and employment constraints (occupational environment), and perceptions regarding the state of awareness of EM and the capacity for change at the societal level (national context).

**Conclusion:**

This work underscores the need to continue to address multiple systemic and cultural issues within the Ethiopian health care landscape in order to address EM graduate retention. It also highlights the potential success of a retention strategy focused on the career ambitions of keen EM doctors.

## Introduction

The global burden of acute illness and injury remains disproportionately placed in low and middle-income countries (LMICs).^1^ Of all trauma-related disabilities and deaths that occur annually worldwide, 90% occur in LMICs,^2^ and evidence suggests that more than one-third of all-causes of death and disability occurring in these settings is related to potentially-treatable health problems.^3^ The World Health Organization attributes this increased morbidity and mortality in LMICs to inadequate infrastructure and medical human resources to provide urgent care to patients following trauma.^4^ Targeted development of emergency care systems remains a regional priority for improving patient outcomes in resource-limited settings across Africa.^5^

Among the challenges encountered in improving emergency care in LMICs, the provision of adequate training and subsequent retention of skilled clinicians are paramount. Across Africa, medical schools struggle to train an adequate number of doctors to meet population needs.^6^–^9^ Physician migration or “brain drain” exacerbates the shortage, as doctors leave to pursue better economic or postgraduate specialty training opportunities abroad.^10^–^12^

In Ethiopia, the effects of medical “brain drain” are profound; between 1987 and 2006, over 70% of the country’s 4,394 doctors either transitioned to private sector practice or emigrated overseas.^13^,^14^ The estimated economic cost of Ethiopian physician emigration exceeds $24 million.^11^ Growing demand for improved access to acute care across Africa has prompted efforts to increase human resource capacity in priority areas such as emergency medicine (EM).

Ethiopia’s commitment to improving the country’s emergency care system includes the development of domestic specialty training opportunities for health care workers. In 2010, Addis Ababa University (AAU) partnered with the University of Toronto and University of Wisconsin to establish the first Ethiopian EM residency program at Tikur Anbessa Specialized Hospital (TASH) in Addis Ababa. Preceding this, there were no formally trained EM specialists in Ethiopia, and no formal emergency care system existed. The program has expanded rapidly. Since 2013, Ethiopia’s first 15 emergency medicine physicians (EPs) graduated from the AAU training program; another 19 post-graduate medical residents (EM residents) were matriculated in early 2016.

As new domestically trained EPs enter the medical workforce in Ethiopia, questions remain around the capacity of the Ethiopian health care system to retain these new graduates as they face expectations to assume positions of leadership for the development of the specialty and the emergency care system itself. This study sought to explore the perspectives of current EM residents, faculty and policy-makers regarding the factors contributing to EP retention in Ethiopia. Specifically, the objective of this study was to describe critical factors that influence the retention of EPs from the perspective of current Ethiopian EM residents, faculty and policymakers in the context of the developing EM system in Ethiopia.

## Methods

### Study design

This was a qualitative study of the perspectives of EM residents, faculty, and policy-makers regarding EP graduate retention in Ethiopia. We used a thematic analysis of in-depth key informant interviews. The study instrument was developed by the study team and pilot tested during the first interviews. No major changes were made to the instruments. Semi-structured face-to-face interviews were carried out by members of the international research team in English with EM residents in October 2012 and with faculty and policy-makers (herein referred to as the stakeholder group) in June 2013. The team members conducting the interviews were members in the TAAAC-EM partnership from Toronto; none were personally responsible for management or employment in the health care system in Ethiopia. The analysis was performed by team members with experience in qualitative participatory research.

### Sampling methods and selection of participants

Twelve key informants were interviewed, including six current AAU EM residents and six stakeholders. A convenience sample of residents was selected to represent the three-year range of the training program (there were no graduates from the program at the time of study). Among the EM residents, one male and one female participated from each year of the program; the average age was 26.5 years (range: 24–31). Stakeholders were recruited based on convenience and purposive sampling in order to represent a range of AAU faculty and administrators and Ministry of Health policy-makers involved in the development of EM in Ethiopia (among the stakeholders, 4 were AAU faculty and 2 were government policy-makers). The average age of stakeholder participants was 41.3 years (range: 25–53) and five of six were male.

### Ethical considerations

This study received ethical approval from the University Health Network Research Ethics Board in Toronto (13-5847-AE, 15-9365-AE) and the Institutional Review Board at AAU (040/13/EM). Residents and stakeholders were invited to participate in the study in person or via telephone, and written consent was obtained before the start of each of each interview.

### Data collection

All resident interviews were conducted by a single moderator (JL). A second moderator (SB) conducted all stakeholder interviews. The moderator administered a structured questionnaire followed by a standard open-ended interview guide (see eSuppl 1). All participants were asked to provide basic demographic, educational, and career history. They were then asked either eight (for residents) or ten (for stakeholders) open-ended questions that sought to elucidate individual perceptions of challenges and opportunities of a future career as an EP in Ethiopia. Preliminary analysis of the interviews revealed sample sufficiency was reached within three interviews from either group.

Interviews lasted 20–75 minutes in length and took place in Addis Ababa. Interviews were digitally recorded, then transcribed verbatim (EF), quality checked by a senior member of the research team (ML), and de-identified prior to analysis.

### Data analysis

Two independent reviewers (AE, MK) who were not involved in the study design developed the coding scheme using inductive methods.^15^,^16^ First, transcripts were unitized into *thought units*. A single thought unit communicates one idea between the interviewer and the participant.^17^ The reviewers applied open codes to the unitized data, then identified patterns within the open codes and developed an initial coding scheme; the coding scheme was refined through an iterative, consensus-based process.^18^ Audit trails were maintained by both reviewers^19^ and discrepancies were resolved by deliberation with a senior member of the research team (ML). The final coding scheme was approved by the research team and a final round of coding was completed applying this scheme to the sample.

The qualitative data were subsequently transformed into quantitative data (see eSuppl 2). Coded thought units were treated as nominal data and a frequency analysis was conducted on the coded data in each transcript.^17^ Response frequency was determined for each factor (sum of number of times an individual factor was mentioned by all participants). A participant count – number of individual participants in each group who mentioned a given factor at least once – was also performed and converted to a participant count ratio for each factor. For example, the factor *financial remuneration* had a participant count of 6 residents and 5 stakeholders; thus, the participant count ratio was 6:5.

Representing findings both qualitatively and quantitatively enriched the analytic process by detecting the frequency and emphasis of particular factors. This strategy allowed us to elucidate particularities between the two participant groups – residents and stakeholders – and to classify factors as convergent or divergent perspectives. We define *convergent perspectives* as those common factors demonstrating the most agreement between the two groups, while *divergent perspectives* were most common factors demonstrating the least agreement between groups. Factors considered *most common* were those factors that had one of the top-ten response frequencies. *Most agreement* was considered a participant count ratio of 4:6, 5:6, 5:5, and 6:6, while a participant count ratio of 0:5, 0:6, 1:5, and 1:6 was considered *least agreement*. In total, 38 factors were identified (see eSuppl 2); ten factors met either participant count ratio criteria or top-ten most common factor ranking criteria, but not both. Twelve factors met both criteria to be considered either *convergent perspectives* (9 factors) or *divergent perspectives* (3 factors), and were the focus of the remainder of this paper (Table 1).

## Results

### Conceptual model

The development of our conceptual model (see eSuppl 3) was informed by three classifications of factors that emerged during coding and subsequent analysis: *individual condition*, *occupational environment*, and *national context*. Factors related to the individual condition affecting EM graduate retention in Ethiopia included personal circumstances and determinants of career satisfaction. Factors regarding the occupational environment focused on systemic issues within the context of practicing EM in Ethiopia, including employment circumstances and financial remuneration for EPs, resource allocation and regulatory mechanisms governing EM as a specialty, and development of the EM residency program. Finally, factors pertaining to the national context encompassed issues of cultural readiness regarding EM in Ethiopian society and the health care system, such as the capacity for change, the strategic climate, and the state of awareness regarding emergency medicine development. The findings discussed here are supported by quotes provided in Tables 1–3.

### Individual condition

Within the concept of individual conditions affecting graduate retention, factors related to career satisfaction were most prominent within both groups of participants. In particular, the potential for career advancement – activities that promote career growth and recognition – emerged as a significant factor affecting career satisfaction in the new EM system. Participants repeatedly identified the opportunity to excel and be rewarded through increasing responsibility and recognition within the Ethiopian academic medical community (Table 1). Residents who expressed specific career ambitions that were related to achieving professorship appeared motivated by teaching opportunities, as in this participant (R1): “If we do specialty they would give us the title of assistant professor. You can teach more, learn more and always take academics to improve your career.”

Motivation to succeed was evident among residents describing a path for their future career. Several participants articulated the impact they envisioned within both medicine and academia. Stakeholders echoed the sentiment that contributions of teaching and scholarship were priorities among residents and would bring positive career satisfaction. Like residents, stakeholders highlighted the career trajectory in university-affiliated hospitals as a draw among new doctors, as articulated by this participant (S5): “And they [the residents] want to teach students, transform students, to becoming doctors. Second, people want to do research and be recognized through research and be promoted in the academic career.”

A disconnect emerged when participants explored the more immediate needs and desires contributing to career satisfaction. EM residents were focused on the relationship between career satisfaction and further opportunities for formal training, such as obtaining subspecialties (i.e. pediatrics, intensive care, and critical care). Most residents were eager to sub-specialize and were willing to travel for this training. A few resident participants acknowledged the benefit that advanced skills and experience accumulated abroad would confer on them as leaders in the developing EM system (R4, R5). In contrast, stakeholders identified tangible individual benefits as key pillars of satisfaction. Recognizing issues of affordability in Ethiopia’s larger cities where many university hospitals are located, stakeholders suggested that provision of housing, transportation, and alternative or non-remunerative mechanisms of compensation would be a significant step toward retaining new EPs in academic centers. While no strategy exists currently to fulfill these needs, stakeholders described several potential options to enable these provisions including building universityaffiliated residential complexes for staff and trainees (S2, S5) and establishment of a government-sponsored tax incentive or credit system to individuals (S6).

### Occupational environment

The discussion of factors attributed to the occupational environment revealed strong consensus across both groups in four themes: EM development, resource allocation, economics, and circumstances of employment. Statements from participants demonstrated a clear motivation and sense of responsibility toward formal support for development of the EM training program and services at AAU (Table 2 in the Appendix). Residents acknowledged that their position as the first graduates of EM in Ethiopia would bestow on them significant teaching responsibility “because emergency medicine needs teachers or regular physicians to teach others” (R1). Some stakeholders who had experienced the birth and development of other medical specialties in Ethiopia described that, along with the sense of responsibility, comes an obligation: “[w]hen a program is growing, especially new, where you cannot transfer [responsibilities] to other people, then really you cannot think of moving elsewhere” (S6), thereby recognizing the how being “pioneers” for a new specialty can transform their responsibility into a burden. Most residents expressed an eagerness to contribute to the duties that will build capacity and strengthen the reputation of EM as a specialty. Residents cited a sense of commitment and ambition toward establishing EM as viable and reputable medical specialty in Ethiopia, taking in stride the simultaneous expectations of “making a society [of EPs] in the nation” (R6).

While perspectives regarding program support were largely positive, economic and resource allocation factors with perceived insufficiency were a negative influence within the occupational environment. A lack of hospital infrastructure and non-human resources were major persistent issues identified among residents and stakeholders. Participants described the frustration of working in “dysfunctional” spaces with incomplete or absent infrastructure and inconsistent supply of equipment and medications (R4). Often working in hospitals without basic emergency medical devices, residents voiced a sense of defeat where they felt unable to provide adequate patient care to those in urgent need: “really you can’t make any difference” (R3).

Participants reflected that these perceived resource deficits challenged the ability of even experienced and highly skilled EPs to care adequately for their patients. Despite a physician feeling sufficiently confident and capable of providing high quality emergency care, one participant (R4) remarked: “Unless you have resources, even if you have knowledge you can’t manage your patients.” Stakeholders acknowledged that working in such poorly resourced environments might dull the enthusiasm among new doctors (S2, S4).

This discussion lead to another point on which the groups converged: the inadequacy of financial remuneration for EPs. Both groups voiced concern that the current salary from the public health care system for emergency physicians is insufficient. Among the resident participants, financial dependence on family was typical: “At this point I’m living with my family and they provide for all my needs but at a certain point if you want to be alone, you don’t earn enough” (R1). Financial remuneration was a driver for medical emigration, which seemed commonplace within both groups of participants. Residents said that many of their colleagues expressed the desire to work abroad “mostly it’s for working to get more money” (R1), while stakeholders quantified the issue on a systemic-level: “Majority [of our medical graduates] are abroad, they are in the United States, Canada, Europe or even African countries. And the main reason is, and the only reason I would say is economical” (S5).

In Ethiopia, the two-tier medical system provides most clinicians with both public and private employment opportunities. One mechanism for mitigating the low salaries in the public health care system is supplementing the physician income via private medical practice. This is a common practice among Ethiopian doctors in other specialties and was described by several participants, including this stakeholder (S6): “… generally speaking, the level of payments in public institution is low. Most of the clinicians are really balancing this by private work and other means.” Stakeholders and residents together expressed concern that given the newness of EM as a specialty in Ethiopia, and the gross lack of infrastructure in hospitals to support emergency care, there is both lack of private demand for EM specialists in the private hospitals and thus a lack of flexibility for these new physicians to improve their economic situation. Participants projected that this economic issue will be problematic throughout the early development of EM (S5, S6).

### National context

Participant responses with respect to the state of awareness and capacity for change characterized a challenging but optimistic trajectory for EM within the national context (Table 3 in the Appendix). Participants acknowledged that EM is emerging into a medical system with a limited awareness of the specialty despite a huge demand for emergency care services, highlighting a need for a unified strategy to elevate the status of EM. Residents cited that in Ethiopia, EM is poorly understood, even among other clinicians (R5, R6). Despite a lack of awareness among their colleagues, residents are confident in their unique abilities as EM clinicians, including those skills that are a vital contribution to the current skillset of Ethiopian physicians. For example, one participant described the distinct competencies with which EM clinicians are equipped, and was optimistic that recognition and understanding would come over time (R6).

Stakeholders expanded the challenges into specific opportunities for change across the system, or cultural needs. Conscious of the state of awareness of EM and a growing demand for emergency care services, stakeholders suggest levying the power of government to advance EM by mandating a key role for EM physicians in operating emergency departments (S3). One stakeholder expressed the imperative for national leadership to ensure the development of EM as Ethiopia develops across a number of important sectors including tourism, finance, and manufacturing (S6).

Identifying system-level change was a point of divergence among the groups, with stakeholders alone describing evidence of shifting policy and procedure at the governmental level that could influence the national environment for EM clinicians. Stakeholders emphasized efforts by the federal government to improve the delivery of emergency care across the country, specifically the institution of a reliable pre-hospital care system and standardization of emergency care infrastructure in hospitals (S4, S6). The scale of such efforts was illuminated through the example of the distribution of more than 800 ambulances to counties with populations of 100,000 or more (S6).

## Discussion

The participants in this study offered a rich dialogue exploring graduate retention in Ethiopia. Our analysis illuminated a web of convergent and divergent perspectives embedded in a conceptual model spanning three domains – the individual condition, occupational environment and national context. At the individual level, career satisfaction figured as an important theme affecting graduate retention, with potential for career advancement being a shared priority among both groups. Residents and stakeholders differed in their prioritization of specific opportunities and benefits that contribute to career satisfaction (further training versus tangible economic benefits), a divergence that likely represents the changing needs of medical professionals over the continuum or maturity of their career. Convergent perspectives were most abundant in the area of the occupational environment, with voices of residents and stakeholders unified across multiple themes and factors: inadequate resources and infrastructure was universally seen as a barrier to everyday functionality of the emergency department, while factors inherent to the two-tiered medical system were perceived as equally problematic in limiting the economic well-being of EPs. While these factors were perceived to negatively influence graduate retention, responsibility to provide support toward the growth and success of the EM program was largely a positive influence. Finally, tension between the need for systemic change and poor understanding of the specialty has created unique and significant challenges for EM clinicians and advocates, and has defined the national context. Nonetheless, key decision-making stakeholders are forging ahead with positive changes across the country to improve the system of emergency care, starting with pre-hospital care.

These results broadly parallel the findings from studies investigating medical emigration from developing countries around the world. Whether the issue is retaining graduates in rural and remote locations, the public health care sector, or maintaining adequate human health care resources across a whole nation, the prominent factors affecting retention of health care workers are common in countries such as Uganda, Nigeria, and Ghana: better training opportunities, higher level of compensation, improved working conditions, hospital set-up and supplies, prospects of promotion and career growth, and greater stability of the political and cultural climate.^10^,^12^,^20^,^21^ While the universality of the situation may seem dire, decision-makers in Ethiopia should look positively at the successful strategies implemented by other national agencies.

The prominence of factors associated with career satisfaction indicates a significant opportunity for Ethiopian decision-makers to leverage non-financial incentives to influence graduate retention. Efforts in Nigeria, Ghana, and other LMICs have capitalized on such incentives to motivate health care workers to stay in rural and remote areas, offering economic subsidies for housing, transportation, and even food, as well as opportunities to acquire advanced training.^12^,^21^–^23^

The benefits to harnessing the ambition of young graduates who are eager for professional development and sub-specialization are manifold. Opportunities for professional development foster growth within a medical specialty and ensure adequate academic capacity to train future clinicians, leading to system improvement and increased satisfaction for physicians.^24^,^25^ Regionally-facilitated professional development could help mitigate the depletion of medical expertise across the continent and the African Federation for Emergency Medicine can play a key role in building regional capacity for systems development.^26^ Sharing lessons and best practices between countries will allow professionals to build networks for educational and research advancements in Africa as EM grows across the continent.^27^

Although inadequate compensation is a prominent theme in many studies, evidence suggests that salary increases may not be an effective means of achieving retention of health care workers in LMICs for several reasons. Destination country economics,^28^ unsustainable models of large-scale wage increases,^29^,^30^ and the role physician values play in determining intent to stay^31^ all threaten the intended outcome. This is good news for decision-makers in Ethiopia, supporting the argument for non-financial incentives. Furthermore, while direct increases in public salary may not be feasible, equitable access to private sector opportunities for EPs should be afforded. Health care leadership and EM advocates need to engage the private sector to provide this important opportunity.

Resource constraints that affect the ability of clinicians to provide quality emergency care to patients contribute to work-related stress, poor job satisfaction and planned reduction of clinical work hours – even in settings where compensation is adequate.^32^,^33^ Enabling EPs access to medical resources, including essential medicines and equipment for the practice of emergency medicine has been highlighted to not only improve patient outcomes but also to support career satisfaction and enhancing training for future generations.^34^ This is an important lesson for the development of EM as a specialty in Ethiopia.

A great challenge facing new EM graduates is that they are entering into a system unfamiliar with EM as a distinct medical specialty. As an emerging specialty in many parts of the world, the role of EM and its practitioners is still being defined and prevailing misconceptions are being dismantled.^5^,^35^,^36^ New EPs and other EM advocates face the obstacle of establishing awareness in both the public and medical communities – a necessary step toward sensitizing the health care system and its users to the value of specialized EM care. Furthermore, an atmosphere of respect and civility among colleagues not only enables multidisciplinary practitioners to provide specialized care to patients in need, but actually contributes substantially to job satisfaction and retention.^37^

Establishing the foundations of a new medical specialty is challenging, no matter the setting. Besides the delivery of quality clinical service, health care systems must account for provision of quality education and training programs, ongoing advancement of the specialty, development of improvement and research priorities, and an administrative framework for sustainability – all within a locally-relevant context.^5^,^35^,^38^–^41^ This level of strategic planning requires commitment from stakeholders at all levels – community-level operational sites, regional regulatory bodies, and policy-makers at the national level.^5^,^39^,^41^ Advocates for the globalization of EM suggest championing of the specialty by a “highly motivated, respected and visionary physician,”^41^ especially someone prominent in another, established medical field, in order to facilitate political commitment for large-scale health care reform.^5^,^39^,^41^ An exemplary success is the establishment of the National Ambulance Service in Ghana in 2004, the result of well-coordinated efforts in the administration, personnel training, operation, and ongoing management of the initiative. With key stakeholders already engaged in the establishment of EM and with relevant examples to follow, this is one area where Ethiopia is well situated to succeed.

There are several existing theoretical models for medical migration. The model developed in this study resembles the work of Franco et al.,^42^ mentioned by Luboga in the context of addressing physician dissatisfaction in Uganda. Franco and colleagues constructed a three-level framework of health worker motivation, with determinants oriented to the personal/internal, work-related, and broad societal contexts.^42^ The framework describes how health sector reform can improve health system performance through positive worker motivation, and prioritizes cultural sensitivity in defining reform strategy, especially in developing countries.^42^ The “push and pull” theory of migration posited by Lee in 1966 is another common framework for discussion of the medical “brain drain” in developing countries,^12^,^31^,^43^ whereby “push factors” encourage emigration from the country of origin and “pull factors” encourage immigration to the destination country. The conceptual model described in this paper addresses perceptions of EM residents, faculty and policy-makers within the unique environment of the Ethiopian health care system, in the context of the beginning of EM as a new specialty. Our work here adds to these two theories in addressing a framework that is contextually appropriate for Ethiopia that includes individual condition, occupational level, and national context factors, and explores push and pull factors within that framework.

### Study limitations

Numerous factors could affect the analysis and interpretation of the findings.

This study addressed only EP retention, and not all aspects of emergency care, and thus should not be interpreted as a comprehensive guide to establishing an emergency care system.

The inclusion of participants in this study may be biased by the voluntary nature of recruitment and the one-on-one interview strategy. All interviews were conducted in English with an interviewer who was a member of the international research team, not a local researcher.

The pool of resident candidates was relatively small (17 residents). The investigators mitigated underrepresentation by including a large proportion of potential candidates. All participants were individuals who were generally interested in the advancement of emergency medicine in Ethiopia in the public health system; no relevant biases were identified among participants.

The evidence-informed qualitative approach^17^ was selected to systematize a descriptive analysis of the findings and to facilitate rich interpretation of the data. This method may have over- or underestimated the importance of any given factor.

### Conclusion

The specialty of emergency medicine is developing in the Ethiopian medical system with limited professional awareness, great demand for service across the country, and a small but determined group of champions including EPs, academics, administrators and policy-makers. While the authors recognize that training and retaining EPs is just one aspect of establishing an effective emergency care system, the detrimental effects of migration of highly skilled medical human resources from LMICs have been previously and thoroughly demonstrated.^11^ This study highlights a complex relationship of factors influencing graduate retention. At the occupational and national levels, multiple systemic and cultural issues challenge graduates’ will and intention to stay. However, an appetite for career growth and contribution to the specialty at the individual level represents a potential opportunity for focused retention efforts to overcome barriers and retain this valuable human resource.

Ethiopia is at the forefront of EM development as a specialty and system in Africa. The high burden of morbidity and mortality from acute illness and injury demonstrate a clear need for effective emergency care, but improvement and innovation remains a challenge in a resource-limited setting unaccustomed to prioritizing acute care. AAU, at Tikur Anbessa Specialized Hospital, is the national leader in emergency system development, including pre-hospital care, hospital restructuring, and building human resources capacity in EM. Graduate retention is a crucial step toward maintaining a sustainable cohort of EPs to staff local emergency departments, to train future generations of EPs, and to lead the evolution of the emergency care system. As the first EPs matriculate from AAU and begin their careers, this study highlights that while challenges exist in retaining these new doctors, retention strategies with demonstrated success in similar settings as well as initiatives unique to the Ethiopian context provide optimism for new EM doctors and stakeholders in this developing emergency care system.

## Supplementary Information







## Figures and Tables

**Table 1:** Summary of quantitative data for twelve factors meeting criteria for *convergent* or *divergent perspectives*

	Factor	Response frequency		Rank*	Participant count ratio

		Residents	Stakeholders		(R:S)
**Convergent perspectives**	Non-human resources	22	22	1 R / 8 S	5:6

	Financial remuneration	20	28	2 R / 3 S	6:5

	Flexibility	16	19	4 R	6:6

	Career advancement	16	17	4 R	6:4

	Professional awareness	15	20	6 R	6:6

	Private demand	13	13	7 R	5:6

	Program support	10	23	8 R / 7 S	5:6

	Culture of change – needs	9	25	9 R / 5 S	6:6

	Infrastructure	7	21	10 S	5:6

**Divergent perspectives**	Training opportunity	20	2	2 R	6:1

	Job benefits	3	23	7 S	1:5

	System-level change	1	29	2 S	1:5

Table legend

* As per the criteria for convergent or divergent perspectives, only factors within the top-ten ranking in either group appear in this table.

R – resident group; S – stakeholder group.

## Appendix

**Table 2:** Supportive quotes for critical findings within the *individual condition*.

*Theme: Factor (Perspective Classification)*	Representative quotes from residents	Representative quotes from stakeholders
*Career Satisfaction: career advancement (convergent)*	Academically I want to do a lot of research that can change the living status and the physical health status of my society. ... I want to learn more and then I want to transfer for generations what I know so I want to go forward to learn subspecialties. (R2) If we do specialty they would give us the title of assistant professor. …You can teach more, learn more and always take academics to improve your career. (R1)	And they [the residents] want to teach students, transform students, to becoming doctors. Second people want to do research and be recognized through research and be promoted in the academic career. So it basically because they have interest in academic medicine, research and service that’s why people are here [at a teaching hospital]. And also, I have to be honest to you that by working here, people are recognized by the public or by other people because being an employee of a university hospital such as this it means something big in its own. (S5)
*Career Satisfaction: training opportunity (divergent), job benefits (divergent)*	I wanted to do some pediatric training separate as an emergency pediatrician. (R1) If I could make it I want to study intensive care. (R3) In addition to the specialty I would like to have also critical care. …We don’t care whether it’s in Africa, Europe or the US, but we need to see what’s going on outside from Addis because there’s no experienced persons here. We need to see how they are functioning and improve the system that we have. We need something new from others. But we want to go somewhere like – who has very experienced knowledge and who has a long history of emergency medicine. (R4) For the time being probably I will go into… Like, I am thinking of critical care medicine (R5) Like for example, I’m thinking of doing endoscopic procedures if they are available. Life-saving procedures. … It’s not available, currently [in Ethiopia]. I think they are available outside, if I had the opportunity I would. (R5)	People who are working in the federal hospitals, we have created a mechanism where they can get some non-financial incentives, like providing house. Addis Ababa University is also trying to follow that system. (S2) Housing is extremely important in Addis. It’s a big problem, so for our young faculty, what the university is thinking is to support staff in providing housing. … some transport facilities, and in the long term, we are planning to build residential complexes for our students, residents and staff. (S5) For example, one idea is that young doctors should have a place to live therefore the government can provide a house or land for construction. These junior doctors need vehicles and vehicles are really expensive. Therefore the government can arrange for some sort of credit system or tax-free system. (S6)

**Table 3:** Supportive quotes for critical findings within the *occupational environment*.

*Theme: Factor (Perspective Classification)*	Representative quotes from residents	Representative quotes from stakeholders
*Program Development: program support (convergent)*	…because emergency medicine needs teachers or regular physicians to teach others. (R1) …it is going to be almost we are making a society [of emergency medicine doctors] in the nation from Black Lion from other hospitals; we are the one for the nation. (R6)	When a program is growing, especially new, where you cannot transfer [responsibilities] to other people. (S6) In fact, more is needed from residents who are going back to other institutions. Because there emergency medicine as an institute or program is not established. Therefore, they have to be pioneers and work with others clinicians to establish a nucleus or taskforce which would work in emergency medicine … (S6)
*Resource Allocation: infrastructure (Convergent), non-human resources (Convergent), financial remuneration (Convergent)*	You can see how it’s dysfunctional to work here. There’s no setup, there’s no suction, there’s no ECG. It’s very frustrating to work here. It’s very, very hard. (R4) Even if the best hospitals in the nation they don’t have adequate set up and consistent supply of equipment and medications. So these things are really frustrating… (R6) That’s most important thing … you have patients with [ventricular tachycardia] and if there is no defibrillator and you have patients in respiratory failure and you don’t have a... mechanical ventilator, really you can’t make any difference. (R3) As far as salary is concerned, physicians are not paid well, like you do a lot and what you earn is like small and that’s pretty much a fact in our country…at this point I’m living with my family and they provide for all my needs but at a certain point if you want to be alone, you don’t earn enough… (R1) But here, I know that every different position is very low paid, but emergency physicians should be paid more even from other positions because the working load is very high. (R4 )	The majority of the physicians are dissatisfied not because their income is low, but the working environment, specifically the infrastructure, the medical equipment, the drug availability and the supplies and so on… it might not be available. Sometimes you know this[sic] emergency physicians must have the passion to save the life of people who are have some emergent difficulty. So if they cannot get the required medical equipment to save this life, they may feel some sorts of frustration. (S2) The emergency department in every hospital of our country is not well equipped, it’s not, the infrastructure is not completed so even if they want to work with what they learned, what they trained, there is no equipment. So even that makes some to be frustrated. (S4) Majority [of our medical graduates] are abroad, there are in the United States, Canada, Europe or even African countries. And the main reason is, and the only reason I would say is economical. Just people want, make a better life, they think there is a better life abroad. The pay is high and so on. (S5)
***Employment Circumstances****: private demand (Convergent), flexibility (Convergent)*	From all the old consultants, like whatever field they are working the one thing they do is they always have to do a private practice to augment their incomes because that’s where you get paid more. Working just in an academic center you won’t earn a lot. (R1) It’s economical issue, the physician is not well paid in general. And when it comes to emergency physician you don’t have a chance to work in private clinic where you can make much money. (R6)	Well, the graduates of emergency medicine will be paid the same as any other professional or faculty… The basic salary for teaching and clinical services. Now, as I told you earlier, this is not enough, that’s why people are doing moonlighting private and so on. (S5) In my opinion, generally speaking, the level of payments in public institution is low. Most of the clinicians are really balancing this by private work and other means. Emergency medicine is a new discipline, these wings are not developed. So for a new graduate, until graduates just develop different angles, different projects or activities, I think the public institutional level payment is low. That is really a challenge area. (S6)

**Table 4:** Supportive quotes for critical findings within the *national context*

*Theme: Factor (Perspective Classification)*	Representative quotes from residents	Representative quotes from stakeholders
*State of Awareness: professional awareness (convergent);*	Yes, we are starting, we are starting to practice and this way not well recognized in the country. Not many people know what it means [to practice emergency medicine], even the [other medical] professionals working here. And I think currently it is difficult to define. (R5) You know this is lack of understanding, but with time, they will know how influential this work is – the other departments, the other specialties, the students, the residents. And they know our differences, we have differences from other departments: I cannot be the same as an internist because I have more skills. More anesthesia skills, more airway skills which is not common in other departments, so they are now identifying differences and through time, probably this will change. (R6)	Emergency cases are prevalent in Ethiopia. That is why the Ministry was talking about the need to increase the human capacity working in emergency services. As far as these people are able to manage the cases ...that is highly recognized …the need based on the perception that they will serve the public ... is highly recognized definitely. (S2)
*Capacity for Change: cultural need (convergent)*	I think that we are going to work in the future on that organization of EMS. Not only me but all of us. All Emergency Medicine doctors, we should work on that. Just to see patient care from the scene of emergency, of illness, maybe trauma or medical illness, to give patients necessary EMS as early as possible. To transport trauma patients to hospital - that will be what we will work in the future. (R3)	Especially on the private sector, the Ministry has to declare that especially big, multi-disciplinary hospitals has to enroll emergency physicians for their emergency units. This has to be clearly stated. … the emergency department has to be run by emergency physician with such services with such activities. If it is declared, this will be a good opportunity. (S3) On the national level, from the government [there] is just great need to get an emergency system. Because in addition to its citizens, the government has a lot of guests. This is a diplomatic centre for Africa with a lot of conferences and I think that the number of tourists has started to grow… investment and industrialization, although gradual, is growing at this time. Therefore it appears there is a great need for emergency medicine development. Although the systems are not strong, there is a great demand from the nation. (S6)
*Capacity for Change: system-level shift (divergent)*	--	In the past years, especially starting from our millennium, Ethiopian millennium [September 11, 2007], we started to focus as Ministry of Health, as Ministry of Health on emergency services and after that even the structure of hospital emergency service and trying to strengthen the pre-hospital service. ….. Currently we are dealing with other partners so that we can equip every emergency department in every hospital. We prepare a standard for emergency room on the structure, on the equipment, on the drug … every hospital should fulfill that standard. We are working with that as a Ministry and working with other partners to fulfill that standard. (S4) In the country, the federal ministry of health now they have distributed about 820 ambulances, one to each small county with a small population of 100 000. … now our taskforce and emergency medicine department has helped the ministry of health to develop [standard] training manuals for ambulance crews, and now we are also supporting the ministry of health in actual training itself. …this is just a huge task because 820 sites with 100 000 population it’s almost serving 82 million, [the whole] population of the country. (S6)

## References

[1] (2008). The Global Burden of Disease: 2004.

[2] Hofman K, Primack A, Keusch G, Hrynkow S (2005). Addressing the growing burden of trauma and injury in low- and middle-income countries. Am J Public Health.

[3] Anderson PD, Suter RE, Mulligan T, Bodiwala G, Razzak JA, Mock C (2012). World Health Assembly Resolution 60.22 and its importance as a health care policy tool for improving emergency care access and availability globally. Ann Emerg Med.

[4] (2010). Strengthening care for the injured: success stories and lessons learned from around the world.

[5] Calvello E, Reynolds T, Hirshon JM (2013). Emergency care in sub-Saharan Africa: Results of a consensus conference. AfJEM.

[6] Mullan F, Frehywot S, Omaswa F (2011). Medical schools in sub-Saharan Africa. Lancet.

[7] Deressa W, Azazh A (2012). Attitudes of undergraduate medical students of Addis Ababa University towards medical practice and migration, Ethiopia. BMC Med Educ.

[8] Chen C, Buch E, Wassermann T (2012). A survey of Sub-Saharan African medical schools. Hum Resour Health.

[9] Greysen SR, Dovlo D, Olapade-Olaopa EO, Jacobs M, Sewankambo N, Mullan F (2011). Medical education in sub-Saharan Africa: a literature review. Med Educ.

[10] Astor A, Akhtar T, Matallana MA (2005). Physician migration: views from professionals in Colombia, Nigeria, India, Pakistan and the Philippines. Soc Sci Med.

[11] Mills EJ, Kanters S, Hagopian A (2011). The financial cost of doctors emigrating from sub-Saharan Africa: human capital analysis. BMJ.

[12] Hagopian A, Ofosu A, Fatusi A (2005). The flight of physicians from West Africa: views of African physicians and implications for policy. Soc Sci Med.

[13] Berhan Y (2008). Medical doctors profile in Ethiopia: production, attrition and retention. In memory of 100-years Ethiopian modern medicine & the new Ethiopian millennium. Ethiop Med J.

[14] Alem A, Pain C, Araya M, Hodges BD (2010). Co-creating a psychiatric resident program with Ethiopians, for Ethiopians, in Ethiopia: the Toronto Addis Ababa Psychiatry Project (TAAPP). Acad Psychiatry.

[15] Choo EK, Garro AC, Ranney ML, Meisel ZF, Morrow Guthrie K (2015). Qualitative Research in Emergency Care Part I: Research Principles and Common Applications. Acad Emerg Med.

[16] Ranney ML, Meisel ZF, Choo EK, Garro AC, Sasson C, Morrow Guthrie K (2015). Interview-based Qualitative Research in Emergency Care Part II: Data Collection, Analysis and Results Reporting. Acad Emerg Med.

[17] Auer-Srnka KJ, Koeszegi S (2007). From Words to Numbers: How to Transform Qualitative Data into Meaningful Quantitative Results. Schmalenbach Business Review.

[18] Hsieh H-F, Shannon SE (2005). Three Approaches to Qualitative Content Analysis. Qual Health Res.

[19] Rodgers BL, Cowles KV (1993). The qualitative research audit trail: a complex collection of documentation. Res Nurs Health.

[20] Luboga S, Hagopian A, Ndiku J, Bancroft E, McQuide P (2011). Satisfaction, motivation, and intent to stay among Ugandan physicians: a survey from 18 national hospitals. Int J Health Plann Manage.

[21] Awofeso N (2010). Improving health workforce recruitment and retention in rural and remote regions of Nigeria. Rural Remote Health.

[22] Hongoro C, Normand C, Jamison DT, Breman JG, Measham AR (2006). Health Workers: Building and Motivating the Workforce. Disease Control Priorities in Developing Countries.

[23] Harries AD, Zachariah R, Bergstrom K, Blanc L, Salaniponi FM, Elzinga G (2005). Human resources for control of tuberculosis and HIV-associated tuberculosis. Int J Tuberc Lung Dis.

[24] Germa F (2011). The development of emergency medicine in Ethiopia. CJEM.

[25] Lalloo UG, Bobat RA, Pillay S, Wassenaar D (2014). A strategy for developing future academic leaders for South Africa in a resource-constrained environment. Acad Med.

[26] Bruijns SR, Wallis LA (2011). Africa should be taking responsibility for emergency medicine in Africa. AfJEM.

[27] Hobgood C, Anantharaman V, Bandiera G (2010). International Federation for Emergency Medicine model curriculum for medical student education in emergency medicine. Emerg Med J.

[28] Vujicic M, Zurn P, Diallo K, Adams O, Dal Poz MR (2004). The role of wages in the migration of health care professionals from developing countries. Hum Resour Health.

[29] Kumar P (2007). Providing the providers - remedying Africa’s shortage of health care workers. N Engl J Med.

[30] Ruwoldt P, Perry S, Yumkella F, Sagoe K (2007). Assessment of the Additional Duties Hours Allowance (ADHA) Scheme: Final Report.

[31] Kotzee TJ, Couper ID (2006). What interventions do South African qualified doctors think will retain them in rural hospitals of the Limpopo province of South Africa?. Rural Remote Health.

[32] Rondeau KV, Francescutti LH (2005). Emergency department overcrowding: the impact of resource scarcity on physician job satisfaction. J Healthc Manag.

[33] Crook HD, Taylor DM, Pallant JF, Cameron PA (2004). Workplace factors leading to planned reduction of clinical work among emergency physicians. Emerg Med Australas.

[34] Cox M, Shao J (2007). Emergency medicine in a developing country: experience from Kilimanjaro Christian Medical Centre, Tanzania, East Africa. Emerg Med Australas.

[35] Anderson P, Petrino R, Halpern P, Tintinalli J (2006). The globalization of emergency medicine and its importance for public health. Bull World Health Organ.

[36] Clarke ME (1998). Emergency medicine in the new South Africa. Ann Emerg Med.

[37] Blaauw D, Ditlopo P, Maseko F (2013). Comparing the job satisfaction and intention to leave of different categories of health workers in Tanzania, Malawi, and South Africa. Glob Health Action.

[38] Anthony DR (2011). Promoting emergency medical care systems in the developing world: weighing the costs. Glob Public Health.

[39] Curry C (2008). A perspective on developing emergency medicine as a specialty. Int J Emerg Med.

[40] Hsia R, Razzak J, Tsai AC, Hirshon JM (2010). Placing emergency care on the global agenda. Ann Emerg Med.

[41] Smith J, Haile-Mariam T (2005). Priorities in global emergency medicine development. Emerg Med Clin North Am.

[42] Franco LM, Bennett S, Kanfer R (2002). Health sector reform and public sector health worker motivation: a conceptual framework. Soc Sci Med.

[43] Akl EA, Maroun N, Major S (2007). Why are you draining your brain? Factors underlying decisions of graduating Lebanese medical students to migrate. Soc Sci Med.

