# Supplementary material for: Examining critical factors affecting graduate retention from an emergency training program in Addis Ababa, Ethiopia: a qualitative study of stakeholder perspectives
Source: Can Med Educ J. 2017 Apr 20;8(2):e61–74. (PMC5669294)
Supplement: Supplementary file 1 [file CMEJ-08-61-eSuppl_1.pdf]

Additional file 1. Survey Tool

RESIDENTS

Demographics/Career history

1. What is your Age: _______________ (years)
2. What is your Sex: (male) or (female)
3. Marital status? (married) or (single)
4. On graduation from residency, do you have a commitment back to a sponsoring hospital or city? (yes) or (no)
    a. If yes, for how many years: _____________
    b. Central/Rural location:
5. What year are you in your residency program: _____________
6. Do you or have you worked in the private sector? Public sector?
7. Have you completed residency training other than EM? (Describe)
8. Have you completed any training abroad? (Describe)

Open-ended questions

1. What do you value in a future medical career?
2. What opportunities do you think will be available for you when you finished residency?
    a. What are your intentions for future employment?
3. What is your understanding of the financial remuneration system now for EM graduates?
4. For the previously identified employment opportunities, do you feel prepared by your EM training program to take on these roles?
5. Out of your medical school class, what percent of MDs have left Ethiopia? If yes, where are they leaving to?
6. What do you think your colleagues' intentions are for future employment after residency training?
7. What do you think the status of EM is currently in Ethiopia?
8. What do you think your role is in the advancement of Ethiopian emergency medical care?
    a. Do you feel that you wish to be a leader of EM in Ethiopia?

STAKEHOLDERS

Demographics/Career history

1. What is your Age? _______________ (years)
2. What is your Sex? (male) or (female)
3. Marital status? (married) or (single)
4. How long have you worked in Ethiopia? # years?
5. What roles have you held previous to and including your current job? (location, years, positions)
6. Where did you complete your education/training? (location and years abroad, years in Ethiopia)
7. Have you ever worked outside of Ethiopia? (location, years and position)
8. What is your specific role in the development of EM at AAU?

Open-ended questions

1. What do you think is most valued by physicians who remain working at AAU or elsewhere within the public system?
2. What opportunities do you think will be available for EM residents when they graduate?

3. What is your understanding of the financial remuneration system now for EM graduates?
4. What are some of the non-financial incentives that can be used to retain graduating EM physicians? What do you think could be improved in this area?
5. What do you think of medical emigration?
6. What do you think the residents' intentions are for future employment after residency training?
7. What do you think the status of EM is currently in Ethiopia?
8. Do you think that a strong EM system in Ethiopia will influence graduate retention?
9. Do you think EM graduates and residency leaders can engage effectively with the government to improve the system? How?
10. What do you think your role is in the advancement of Ethiopian emergency medical care?
    a. Do you wish to be a leader in the development of EM as a specialty in Ethiopia?
