# Supplementary material for: Examining critical factors affecting graduate retention from an emergency training program in Addis Ababa, Ethiopia: a qualitative study of stakeholder perspectives
Source: Can Med Educ J. 2017 Apr 20;8(2):e61–74. (PMC5669294)
Supplement: Supplementary file 2 [file CMEJ-08-61-eSuppl_2.pdf]

Additional file 2. Resident and stakeholder responses quantified by frequency, rank and participant count.

	Factor	Response frequency: Residents	Response frequency: Stakeholders	Rank (top 10 only) *	Participant count ratio (R:S)	R1	R2	R3	R4	R5	R6	S1	S2	S3	S4	S5	S6
Individual condition	*THEME: Personal circumstances*																
	Self & cultural identity ‡	8	20	--	4:6		x	x	x	x		x	x	x	x	x	x
	Social responsibility	5	11	--	3:5		x	x		x		x	x		x	x	x
	Responsibility to grow specialty ‡	9	3	9 R	6:2	x	x	x	x	x	x				x		x
	Social influence	4	7	--	2:4		x			x		x	x	x			x
	THEME: Career satisfaction																
	**Career advancement +**	16	17	4 R	6:4	x	x	x	x	x	x	x		x		x	x
	**Job benefits †**	3	23	7 S	1:5					x		x	x	x		x	x
	**Training opportunity †**	20	2	2 R	6:1	x	x	x	x	x	x						x
	Fulfillment	2	16	--	2:6	x			x			x	x	x	x	x	x
	Professional development ‡	7	10	--	5:5	x	x	x	x		x	x		x	x	x	x
	Workplace environment ‡	9	19	9 R	3:5				x	x	x		x	x	x	x	x
Occupational environment	*THEME: Employment circumstances*																
	**Private demand +**	13	13	7 R	5:6		x	x	x	x	x	x	x	x	x	x	x
	**Flexibility +**	16	19	4 R	6:6	x	x	x	x	x	x	x	x	x	x	x	x
	Job opportunity‡	6	20	--	4:6		x	x	x	x		x	x	x	x	x	x
	THEME: Economics																
	**Financial remuneration +**	20	28	2 R / 3 S	6:5	x	x	x	x	x	x	x		x	x	x	x
	Source of income	3	13	--	2:5	x					x	x	x	x		x	x
	Regional pay variance	0	4	--	0:2							x					x
	Pay equality ‡	1	7	--	1:5				x			x	x	x		x	x
	Pay equity	2	5	--	2:4	x			x				x	x		x	x
	THEME: Resource allocation																
	**Non-human resources +**	22	22	1 R / 8 S	5:6	x		x	x	x	x	x	x	x	x	x	x
	**Infrastructure+**	7	21	10 S	5:6	x	x	x	x		x	x	x	x	x	x	x
	Pre-hospital care ‡	2	24	6 S	2:6			x			x	x	x	x	x	x	x
	Human resources capacity	6	21	10 S	3:6	x			x		x	x	x	x	x	x	x
	Human resources distribution	0	3	--	0:2								x	x			

	THEME: Regulatory mechanisms																
	Return to service obligation	6	16	--	4:5	x		x	x		x	x	x	x	x		x
	Service restriction	6	5	--	4:3	x			x	x	x	x		x			x
	Professional standards	0	6	--	0:2							x					x
	THEME: Program development																
	**Program support** +	10	23	8 R / 7 S	5:6	x	x	x		x	x	x	x	x	x	x	x
	Program commitment	3	27	4 S	3:6	x			x		x	x	x	x	x	x	x
	Program growth	2	13	--	2:6				x	x		x	x	x	x	x	x
National context	*THEME: State of awareness*																
	**Professional awareness** +	15	20	6 R	6:6	x	x	x	x	x	x	x	x	x	x	x	x
	Public awareness ‡	6	17	--	5:6	x	x		x	x	x	x	x	x	x	x	x
	THEME: Capacity for transformation																
	**System-level change** †	1	29	2 S	1:5						x		x	x	x	x	x
	**Culture of change - needs**+	9	25	9 R / 5 S	6:6	x	x	x	x	x	x	x	x	x	x	x	x
	Culture of change – progress ‡	4	33	1 S	3:6		x			x	x	x	x	x	x	x	x
	THEME: Strategic climate																
	Advocacy	6	17	--	3:4			x	x		x	x		x	x	x	
	Policy & decision-making‡	0	16	--	0:5							x		x	x	x	x
	Government regulation	2	12	--	2:6				x		x	x	x	x	x	x	x
	Political intention	2	6	--	2:4		x				x	x			x	x	x

Table legend
* R – resident group; S – stakeholder group
+ Factor in bold meets convergent perspective criteria (factor with most agreement, as determined by a participant count ratio of 4:6, 5:6, 5:5, or 6:6, and a ranking within the top ten most common factors by response frequency within either group).
† Factor in bold meets divergent perspective criteria (factor with least agreement, as determined by a participant count ratio of 0:5, 0:6, 1:5, or 1:6, and a ranking within the top ten most common factors by response frequency within either group).
‡ Factor meets either participant count ratio criteria OR has top 10 most common factor ranking, but not both.
