# Supplementary material for: Examining critical factors affecting graduate retention from an emergency training program in Addis Ababa, Ethiopia: a qualitative study of stakeholder perspectives
Source: Can Med Educ J. 2017 Apr 20;8(2):e61–74. (PMC5669294)
Supplement: Supplementary file 3 [file CMEJ-08-61-eSuppl_3.pdf]

Additional file 3.

Detailed conceptual model, including concepts, themes and individual factors affecting graduate retention in emergency medicine in Ethiopia.

Individual condition

Career satisfaction

- Fulfillment
- Career advancement
- Professional development
- Job benefit
- Work environment
- Training opportunity

Personal circumstances

- Social influence
- Self & cultural identity
- Social responsibility
- Responsibility to grow specialty

Occupational environment

Program Development

- Program growth
- Program support
- Program commitment

Resource allocation

- Non-human resources
- Human resources distribution
- Human resources capacity
- Infrastructure
- Pre-hospital care

Economics

- Financial remuneration
- Source of income
- Regional pay variance
- Pay equality
- Pay equity

Employment circumstances

- Job opportunity
- Private demand
- Flexibility

Regulatory mechanisms

- Return to service obligation
- Service restriction
- Professional standards

National context

State of awareness

- Public awareness
- Professional awareness

Capacity for change

- System-level change
- Cultural need
- Cultural progress

Strategic climate

- Advocacy
- Policy & decision-making
- Government regulation
- Political intention
